# Will Natural Media Make Online Physicians More Trustworthy? The Effect of Media Naturalness on Patients' Intention to Use HIT

**DOI:** 10.3389/fpsyg.2022.878573

**Published:** 2022-05-26

**Authors:** Shuting Xiang, Weiru Chen, Banggang Wu, Dan Xiang, Shan Wu

**Affiliations:** ^1^School of International Business, Southwestern University of Finance and Economics, Chengdu, China; ^2^Business School, Sichuan University, Chengdu, China

**Keywords:** media naturalness theory, intention to use HIT, communication effort, communication ambiguity, trust toward online physicians

## Abstract

Although previous studies have recognized the important role of patients' trust in promoting their intention to use health information technologies (HIT), most of those studies were under the “risk-benefit” theoretical framework. To deepen the understanding of patients' online consultation decisions, this paper develops a dual-path model investigating how patients develop trust beliefs toward online physicians from the perspective of communication. Drawing on media naturalness theory, we propose that HIT media naturalness will improve patients' perception of communication effort from online physicians and decrease communication ambiguity between patients and online physicians. This improved communication will further strengthen patients' trust in online physicians and promote their intention to use HIT. Based on a two-wave time-lagged survey from 361 participants, the empirical results demonstrated that the relationship between HIT media naturalness and patients' intention to use HIT is individually and serially mediated by two chains, including (1) perceived communication effort and patients' trust and (2) perceived communication ambiguity and patients' trust. We thus contribute to the related literature and provide practical implications.

## Introduction

With the increasing attention to and demand for health information, online health care platforms have emerged as an important channel for individuals to obtain medical knowledge via the internet. The platforms provide patients with geographic convenience (Zhang and Zhang, 2021), online health consultation services (Wan et al., 2020), and good privacy protection (Zahedi et al., 2016). Although customers have recognized the advantages of online health care platforms, not all of them are willing to use these platforms. Thus, a large number of scholars have studied what would affect customers' intention to use health information technologies (HIT), which can be defined as the patients' willingness to receive health care services online, for example, through health care websites, remote consultations, virtual health communities, and so on (Slepchuk et al., 2021). Empirical evidence has shown that the characteristics of online health care platforms (i.e., Yang et al., 2018; Xie et al., 2020), patients' perception of HIT and online physicians (i.e., Le et al., 2019; Kokkoris and Kamleitner, 2020; Zhang and Zhang, 2021) and patients' attitudes (Li D. et al., 2020) affect patients' intention to use HIT.

According to the prior studies, trust toward online physicians has been demonstrated as an extremely important factor influencing patients' willingness to use HIT (Chen and Dibb, 2010; Li et al., 2018; Wan et al., 2020; Gong et al., 2021; Slepchuk et al., 2021; Yang et al., 2021). However, most of the extant studies revealing how patients develop trust beliefs were based on a risk-benefit framework (Fang et al., 2014; Kim, 2014; Ou et al., 2014; Sollner et al., 2016; Venkatesh et al., 2016; Yang et al., 2021). For example, Hong et al. (2019) showed that perceived risk was negatively related to patients' online trust; while perceived benefit was positively associated with patients' online trust. Little research has investigated how patients develop trust from other perspectives or theories. To deepen the understanding of patients' online healthcare continuance decisions, Zahedi et al. (2016) proposed a new design based on media naturalness theory (MNT) (Kock, 2004, 2009), pointing out the important role of media design features in influencing patients' trust and intention to use HIT in the digital context. Media's naturalness refers to the degree of similarity of the medium to the face-to-face communication, and MNT argues that high levels of media naturalness would reduce communication obstacles. Yang et al. (2021) also suggested that future studies could investigate the cognitive mechanism of customer behavior intention based on MNT. Responding to this call, this study aims to explore how characteristics of online healthcare platform, more precisely, media naturalness would affect patients' trust and their further intention to use HIT.

To investigate the mechanism on how media naturalness would influence patients' trust in online physicians and intention to use HIT, we draw on MNT and aim to reveal the internal mechanism from the perspective of communication. This is because media naturalness has been commonly shown to influence actors' perceptions and behaviors through the effectiveness of communication (Kock, 2002, 2005; Kock et al., 2015). Specifically, natural mediums are more likely to provide an immersive environment for patients to communicate with online physicians, which will make it possible for them to convey more detailed and accurate information (Kock, 2002; Zahedi et al., 2016). As a result, on the one hand, patients will be engaged in more communication interactions and perceive online physicians' communication effort; on the other hand, more information and perceived transparency in interaction (Zhang and Zhang, 2021) could help reduce communication ambiguity.

We believe that patients' perceptions of online physicians' communication effort and reduced communication ambiguity will further promote patients' trust in online physicians and enhance their intention to use HIT. This effect occurs because as patients can more readily perceive online physicians' communication effort, they are more likely to believe in the quality of health care information, and therefore trust, online physicians (Yoo et al., 2020). This trust, in turn, improves patients' intention to use HIT. Meanwhile, if less communication ambiguity is perceived in online health care platforms, patients will be more likely to receive and understand health information from online physicians (Zahedi et al., 2016); thus, they will be more likely to trust online physicians and more likely to use HIT.

**Figure 1:**
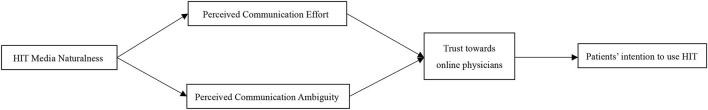
Theoretical framework.

In conclusion, drawing on MNT, our study provides a dual-path model regarding how media naturalness will influence patients' intention to use HIT from the perspective of communication. The theoretical model is shown in Figure 1.

This study offers three key contributions to the literature. First, drawing on MNT, we contribute to the research on patients' intention to use HIT. Existing studies have commonly investigated what would affect patients' behavioral intention from a risk-benefit framework. Responding to the call to explore more cognitive mechanisms (i.e., Yang et al., 2021), this study applies MNT and investigates how HIT media naturalness would influence patients' behavioral intention from the perspective of communication. To our knowledge, this study is the first attempt to develop the association between MNT and the intention to use HIT, which enriches related research fields. Second, although the previous studies have revealed that the characteristics of online platforms play important roles in affecting patients' intention to use HIT (i.e., Yoo et al., 2020; Gong et al., 2021), many more characteristics are worth exploring. By exploring how media naturalness would influence patients' intention to use HIT through a dual-path model, we aim to enrich studies on the antecedents of patients' behavioral intention. Last, by examining how perceived communication effort and communication ambiguity would affect patients' trust toward online physicians, our study also aims to expand the research of patients' trust.

## Theoretical Review and Research Hypotheses

### HIT Media Naturalness and Intention to Use HIT

In a broad sense, HIT is an approach that utilizes cloud-based database tactics to maintain patient related records, information, and reports, so as to properly and effectively handle cases (Singh et al., 2020). In a narrow sense, HIT is the way for patients to receive health services by using healthcare websites, remote consultations, online medical consultation, and virtual health communities (Ni et al., 2020; Slepchuk et al., 2021). Since behavioral intention refers to a person's willingness to perform certain behaviors (Glanz et al., 2015), in this study, we adopted the narrow sense of HIT and define the intention to use HIT as a person's willingness to receive health services online through remote consultations, virtual health communities, health apps or websites, and so on.

Characteristics of the platform have been shown to influence patients' intention to use HIT. For example, the blockchain certificate and structure guarantee provided by the platform would promote users' willingness to use (Xie et al., 2020; Yoo et al., 2020; Shao et al., 2022). These institutional cues can help individuals assess the ability and credibility of the platform, especially for those unfamiliar with it (McKnight et al., 2002; Kim and Lee, 2009). Meanwhile, the previous studies have shown that platform reputation has a significant influence on users' perceived information privacy concerns and their behavioral intention (Eastlick et al., 2006; Gong et al., 2021). Team strength and similarity also have a positive influence on physician trust (Li D. et al., 2020). In addition, Yoo et al. (2020) proposed that when accessing the platform, patients often expect to find a better channel, the functions of which contribute to health information search, doctor selection, health consultation, and rating. As an important characteristic of online platform, media naturalness might be a potential factor influencing patients' intention to use HIT.

Based on the modern human evolution theory, Kock (2002) developed the media naturalness theory. Media naturalness is defined as the degree of similarity of the medium to the face-to-face communication (Kock, 2004). The extent of naturalness can be determined by five factors, including collocation, synchronicity, facial expressions, body language and speech (Kock, 2004, 2005). The higher the extent to which these factors are achieved, the more natural the people will perceive the HIT media to be. For example, people consider e-mail to be more unnatural and less preferable than face-to-face communication (Kock, 2001, 2002). This is because communication media suppresses some key elements in face-to-face communication as the internet is a media of low naturalness (Kock, 2007). The recent technologies have enabled Internet-based medium to provide sufficient information, making it much more similar to natural face-to-face communication than before. This kind of simulated face-to-face medium satisfies the needs of information transmission (Lim and Wollscheid, 2020). With the development of technology, the medium is likely to become increasingly natural.

We propose that the media naturalness of HIT will influence patients' intention to use HIT. First, by showing a high degree of collocation, HIT media naturalness provides an immersive environment for patients (Blau et al.'s, 2017). This will further act as a channel to communicate with online physicians and other patients in communities freely and anonymously. This immersive environment may protect patients' privacy and make them feel comfortable through online communication (Zahedi et al., 2016), thus increasing their intention to use HIT. It is therefore a reasonable expectation that the immersive environment created by the media naturalness of HIT promotes patients' intention to use HIT.

Second, enabling high levels of synchronicity and two-way communication of facial expression and body language, the HIT medium possesses a high level of social presence and promotes patients' intention to use HIT. Specifically, the previous studies have found that social context, online communication and interactivity would influence social presence in the context of virtual consultation (Tu, 2000). With high levels of media naturalness, HIT provides patients with two-way communication, which enables patients and physicians to respond to stimuli immediately and spontaneously (Blau et al.'s, 2017; Shkurko, 2022). Thus, patients will experience high levels of interactivity, which would increase their perceptions of social presence (Tu, 2001; Cui et al., 2013). Moreover, the social presence may elicit patients' emotions and make communication more expressive and engaging (Hwang and Park, 2007), which may thus enhance their intention to use HIT.

Third, the more natural the HIT medium is, the less communication uncertainty during communication, and patients' intention to use HIT will increase. Specifically, facial expression and body language can convey verbal and non-verbal information, which can reduce patients' perception of uncertainty about physicians and the technology (Shi et al., 2020). When patients feel less uncertainty, they are more likely to believe that they will receive accurate, comprehensive and high-quality diagnosis services through this natural communication (Shi et al., 2020). Patients' intention to use HIT may be enhanced under these circumstances. It is reasonable to propose that patients' intention to use HIT will be enhanced through HIT media which provides them with a more natural channel to communicate with others.

In short, HIT media naturalness may enhance patients' intention to use HIT by creating an immersive environment, increasing social presence and reducing uncertainty. Accordingly, we propose Hypothesis 1 as follows:

**H1:** HIT media naturalness will be positively related to patients' intention to use HIT.

### HIT Media Naturalness and Perceived Communication Effort/Communication Ambiguity of Online Physicians

We believe that HIT media naturalness will influence patients' perceived communication effort made by online physicians and their perceived communication ambiguity. Communication effort is one of the dimensions of physicians' benevolence, which refers to physicians' willingness to communicate with patients and make effort to improve their communication skills (Wan et al., 2020). The reduction of media naturalness usually leads to an increase in cognitive effort (Kock, 2002), which is related to patients' perceived physicians' communication effort. We propose that with higher levels of HIT media naturalness, patients are more likely to perceive online physicians' communication effort for the reasons discussed in the following paragraphs.

HIT media naturalness enables patients to clearly understand the information from online doctors in online health services. The high levels of media naturalness provide an immersive environment in which patients can interact with doctors effectively (Zahedi et al., 2016), which can help doctors better display and transmit their health information. In addition, when seeking for the health information, a natural medium always provide patients with doctors' information (such as the doctors' name, professional titles, etc.) (Wan et al., 2020). This information could help to reduce risk perception and uncertainty, improving patients' perception that the health services they receive are of high quality (Manchanda et al., 2015; Marrero et al., 2020). This will also act as a signal to show physicians' communication effort (Gong et al., 2021). When patients recognize that online physicians provide accurate information and reliable services, they would believe that doctors are making a greater effort to communicate.

In addition, HIT media naturalness enables patients to better recognize verbal and non-verbal messages from online physicians during online health services. With a higher level of naturalness, online communication is similar to the ability to interact and respond face-to-face, and patients would perceive the interaction to be more similar with real-world interactions (Klein, 2003). Natural HIT mediums provide virtual places for people to congregate and meet with other people's avatars, so as to create a feeling of being with others (Zahedi et al., 2016). In this case, the communication between doctors and patients becomes more natural, and hence doctors can express their concerns about patients through accurate speech and facial expressions (Kock, 2002). Meanwhile, the communication process with doctors enables patients to obtain enough information to effectively interpret the message being communicated, which is likely to contribute to an increase in perceived physicians' communication effort (Wu et al., 2020). In short, natural HIT enables patients to perceive more information and message from online physicians, so as to enhance their perception of online physicians' communication effort. Accordingly, we propose Hypothesis 2a as follows:

**H2a:** HIT media naturalness will be positively related to patients' perceived online doctors' communication effort.

Communication ambiguity has commonly been defined as the degree of the mismatch between what is trying to be conveyed and what is actually comprehended in communication (Kock et al., 2015). Multiple interpretations will lead to the ambiguity of communication information (Nagasundaram and Wagner, 1992), For example, individuals raised in different cultural environments usually have different information processing schemas, which will lead individuals to interpret information in different ways (Kock, 2002, 2005). We argue that the communication ambiguity is more likely to arise in a low naturalness communication medium. The HIT media naturalness can reduce patients' perceived communication ambiguity in two ways.

On the one hand, a natural medium makes it possible to convey more speech and facial expressions to reduce mismatch, and consequently reduce communication ambiguity. Through online communication, dealing with ambiguity is more problematic because voice and gestures are missing (Hisarciklilar and Boujut, 2009). The improvement of media naturalness allows communication synchronicity and the ability to convey speech and facial expressions (Kock, 2002), which could help reduce communication ambiguity among actors. By contrast, a low natural medium, which represents a poor similarity of the medium with face-to-face communication (Blau et al.'s, 2017), is likely to increase the possibility of misunderstanding of communication clues, thus increasing communication ambiguity (Kock, 2002). Therefore, using a more natural medium for communication would help reduce communication ambiguity (Kock et al., 2015).

On the other hand, users' perception of transparency could help decrease the possibility of communication ambiguity through a natural medium. Through online communication, information asymmetry and information risk are greater due to the lack of face-to-face communication (Gong et al., 2021). With high levels of media naturalness, communication is more similar to face-to-face communication (Kock, 2004). Therefore, media naturalness enhances patients' perception of HIT transparency, which improves the physician–patient relationship and reduces communication ambiguity (Zhang and Zhang, 2021). In short, HIT media naturalness will decrease the possibility of patients' perceived communication ambiguity by conveying speech and facial expressions and enhancing patients' perceived HIT transparency. Accordingly, we propose Hypothesis H2b as follows:

**H2b:** HIT media naturalness will be negatively related to patients' perceived communication ambiguity.

### Communication Effort/Communication Ambiguity and Patients' Trust

Existing studies have commonly focused on patients' trust toward online health service from two dimensions: interpersonal and platform (Xie et al., 2020; Yoo et al., 2020; Gong et al., 2021). The interpersonal trust is mainly reflected in physician–patient interactions, which would be influenced by physicians' characteristics (e.g., benevolence, competence, integrity, etc.) (Li et al., 2018; Wan et al., 2020; Cantarutti and Pothos, 2021; Gong et al., 2021). Platform trust refers to patients' trust toward platform, which would be affected by blockchain certificates and structure guarantees provided by the platform (Xie et al., 2020; Yoo et al., 2020; Shao et al., 2022), platform reputation (Eastlick et al., 2006; Gong et al., 2021) and platform strength (Li D. et al., 2020). In this study, we focus on interpersonal trust from patients, which could be characterized by two dimensions, including cognition-based trust and affect-based trust (McAllister, 1995). The cognitive-based trust is called as “trust from the head” (Chua et al., 2008), which is related to a person's basic characteristics, such as ability and reliability (Dirks and Ferrin, 2002). The affective-based trust is called as “trust from the heart” (Chua et al., 2008), which is based on emotional bonds between members, such as understanding of reciprocal sentiments (McAllister, 1995; Dirks and Ferrin, 2002). Empirical studies have shown that physicians' abilities such as professional knowledge, physician rank, treatment effect and physician image are conducive to patients' cognitive trust, while physicians' integrity and benevolence are conducive to patients' affective-based trust (Wan et al., 2020).

We believe that the perceived high levels of online physicians' communication effort could help develop patients' trust toward online physicians. When patients perceive the high levels of physicians' communication effort, they will believe that the physician on the platform can provide sufficient and useful information, and would perceive the information to be of high quality and, in turn, derive more benefit from the platform (Yoo et al., 2020). Thereafter, patients will be more willing to believe that doctors can address their relevant health problems and will trust the physicians' diagnoses (Zahedi et al., 2016). In this case, communication effort of a physician are crucial in physician–patient communication, which increase patients' cognitive trust. In addition, when patients are aware of doctors' communication effort, it can promote smooth communication between physicians and patients and establish a good relationship between the two (Wan et al., 2020). When engaged in more emotional communication with online physicians, patients will develop more affective trust toward online physicians. In short, patients' perceived online physicians' communication effort will increase the possibility of patients' trust toward online physicians by enhancing their cognitive trust and affective trust. Accordingly, we propose Hypothesis H3a as follows:

**H3a:** Patients' perceived online physicians' communication effort will be positively related to patients' trust toward online physicians.

We propose that patients' perceived communication ambiguity will also influence patients' trust toward online physicians. A perceived communication ambiguity is related to the degree of ambiguity, confusion, and lack of clarity involved in communication tasks (Kock et al., 2015). Communication is essential for building a mutually trusted physician–patient relationship (Bombeke et al., 2011). This is because medical and health services are based on information exchange (Rudawska and Krot, 2018), which requires a clear communication process between physicians and patients. The effectiveness of online medical care depends on the ability of physicians to deliver clear and understandable information (Zahedi et al., 2016). Vague or inaccurate communication often leads to misunderstanding, which may cause serious consequences (Hisarciklilar and Boujut, 2009). Failure to communicate or lack of communication may induce the deterioration of patients' physical disease (Kliszcz, 2000), resulting in their distrust of physicians (Cant, 2009). In short, the increase in communication ambiguity may damage patients' trust toward online physicians due to misunderstanding and misdiagnose. Accordingly, we propose Hypothesis H3b as follows:

**H3b:** Patients' perceived communication ambiguity will be negatively related to patients' trust toward online physicians.

### Trust and Intention to Use HIT

We argue that when trust their online physicians, patients are more likely to use HIT. First, if patients believe that online physicians are a credible source of information, which can provide reliable health information, they will be more likely to believe in the benefits of online health services (Yoo et al., 2020). Meanwhile, rational individuals would act in their own benefits when making the decision to trust (Berg et al., 1995; Lewicki and Bunker, 1995), thus affecting the continuance intention of using online health service (Hong et al., 2019; Yang et al., 2021). Second, trust plays an important role in the adoption of health services because it can eliminate the uncertainty related to the undesirable behavior of providers (Li et al., 2018). Trust toward online physicians will reduce patients' perceived uncertainty about the system and associated processes (Chen and Dibb, 2010), which can help them accept the risks, thereby promote the acceptance and use of HIT (Slepchuk et al., 2021). In short, patients' trust toward online services will promote their intention to use HIT by perceiving credible information and eliminating uncertainty. Accordingly, we propose Hypothesis 4 as follows:

**H4:** Patients' trust toward online physicians will be positively related to their intention to use HIT.

### The Chain Mediating Roles of Communication Effort/Ambiguity and Trust

As discussed here, it is predicted that HIT media naturalness can provide an immersive environment to create a sense of real-time interaction for patients. This interaction can stimulate more accurate communication, which can reduce the cognitive ambiguity in doctor–patient communication and enhance patients' perceived online physician's communication effort (Zahedi et al., 2016). When patients perceived high levels of online physician's communication effort, they would feel that doctors can provide useful information to solve relevant health problems. Then they would perceive greater information quality and benefits (Yoo et al., 2020), thus promoting their trust toward physicians and improving their intention to use HIT. According to the statements above, HIT media naturalness stimulates the cognitive process (perceived online physician's communication effort) of patients, then activates emotional units (trust), and finally affects their behavioral decision-making (intention to use HIT). Accordingly, we propose Hypothesis H5a as follows:

**H5a:** Patients' perceived online physicians' communication effort and trust toward online physicians serially mediate the relationship between HIT media naturalness and patients' intention to use HIT.

According to the media naturalness theory, we also argue that the influence of HIT media naturalness on patients' intention to use HIT is serially mediated by patients' perceived communication ambiguity and trust. Specifically, high level of HIT media naturalness would provide a synchronous environment, which can help online physicians convey accurate speech and facial expressions (Kock, 2002). Then patients' perceived transparency is increased and the likelihood of misinterpretation of communication cues is reduced, thus reducing communication ambiguity. Furthermore, patients' needs for information exchange and satisfactory interpersonal communication can be met when they perceive less communication ambiguity from online physicians (Cant, 2009; Zahedi et al., 2016); thus, promoting mutual trust in the doctor–patient relationship. Furthermore, trust toward physicians can increase patients' recognition of the reliability and predictability of online health services (Cantarutti and Pothos, 2021), thus promoting patients' continuance intention of online health services. Accordingly, we propose Hypothesis H5b as follows:

**H5b:** Patients' perceived communication ambiguity and trust toward online physicians serially mediate the relationship between HIT media naturalness and patients' intention to use HIT.

## Methods

### Participants

A time-lagged survey was designed to test the hypotheses. We applied this time-lagged survey design to alleviate the potential common method bias due to our data collection from single source. According to the suggestions of Podsakoff et al. (2003) suggestion, creating a temporal separation by introducing a time lag between the measurement of independent variables and dependent variables is a way to remedy common method bias. Thus, we followed this suggestion and measured control variables, independent variable and mediators in the first stage at Time 1; and measured mediators in the second stage, as well as our dependent variable at Time 2. In addition, the time-lagged survey design has been shown to outperform the cross-sectional design because it can examine causation over time (Tims et al., 2016). By collecting the variables in sequence with a time lag, we can prove the causal effect between the independent variables and dependent variables.

We hired respondents and distributed questionnaires *via* Credamo, a reliable online Chinese data collection platform (www.credamo.com). The respondents were completely anonymous in the process of filling in the questionnaires and obtained certain material reward upon completion. We invited the respondents to participate in our time-lagged survey with a one-week interval. At Time 1 (T1), we hired 429 participants and asked them to rate all control variables (gender, age, education and pay level), HIT media naturalness, communication ambiguity, and communication effort. A week later, those who responded at T1 were asked to complete the Time 2 (T2) survey, which included items measuring trust and intention to use HIT, and we obtained 362 responses (response rate = 84.38%). After matching survey data from two time periods, we eventually obtained 362 responses. After sorting out an invalid questionnaire with missing information on age, 361 samples (*N* = 361) were finally included in our empirical analysis. As for gender, more than half the respondents (60.9%) were women and 91.7% of the participants were aged between 20 and 40 years. Regarding the education level, the proportion of undergraduates reached 71.7%. In terms of pay, 41.6% of the respondents' annual income was between 100,000 and 200,000 yuan in RMB.

According to Armstrong and Overton's (1977) suggestion, we tested non-response bias by comparing respondents with non-respondents in terms of control variables (e.g., in Chang, 2017; Ren et al., 2022). The results of *t*-statistics for the two groups showed no significant differences in age, pay and education level (*p* > 0.05), but a significant difference in gender (*p* < 0.01). To further examine whether this non-response bias from gender would influence our research results, we compared respondents with non-respondents in terms of independent variable (HIT medium naturalness) and mediators (communication ambiguity and communication effort). The results demonstrated that there were no significant differences (*p* > 0.05). The results in the regression section will also prove that gender will not be an impactful factor in regression, thus showing that non-response bias is not a serious issue in our study.

### Measures

All measures used in this study have been validated in the previous research. Given that all administered items were in Chinese, translation and back-translation procedures were followed to ensure the quality of translations (Brislin, 1986). Each measure used a 5-point Likert-type scale ranging from “strongly disagree” to “strongly agree.”

**The HIT media naturalness (T1):** Measures of media naturalness were developed based on Blau et al.'s (2017) definition of HIT media naturalness. In the work of Blau et al.'s (2017), they identified five criteria for assessing the level of media naturalness, including co-location, synchronicity, and the possibility of identifying and conveying facial expressions, body language, and natural speech. We thus developed a five-item scale accordingly (Cronbach-α = 0.828). Instead of measuring the objective natural degree of one specific HIT, we measured respondents' perceived media naturalness based on their past HIT use experience. The respondents might have used different kinds of HITs, so they were able to give an average assessment for the HITs they have used, evaluating the average degree of similarity of those HITs to the face-to-face communication. This approach is consistent with our definition of media naturalness, which characterizes the degree of similarity of the medium to face-to-face communication.

An example item was “please recall your past experience of online health service, would you agree that the communication between you and your online physicians has high levels of synchronicity, which enabled immediate and spontaneous responses to stimuli?” The full questionnaire of HIT media naturalness we developed in this study is in the Appendix. The results suggested a satisfactory internal consistency reliability (CR) of items (which is equal to 0.830), and good validity (all factor loadings > 0.5; χ^2^*/df* = 3.112, RMSEA = 0.077, SRMR = 0.021, CFI = 0.991, IFI = 0.991).

**Communication ambiguity (T1):** For communication ambiguity, the three-item scale was adapted from Kock et al. (2015) and their measure (Cronbach-α = 0.826). An example item was “please recall your past experience of online health consultation, would you agree that the communication between you and online physicians has often been ambiguous?.”

**Communication effort (T1):** Communication effort were measured with those of Wan et al. (2020) and their three-item scale (Cronbach-α = 0.757). An example item was “please recall your past experience of online health consultation, would you agree that online physicians can understand your needs and interact with you effectively?”

**Trust (T2):** To assess patients' trust toward online physicians, we used the measure of Wan et al. (2020). The seven-item measure (Cronbach-α = 0.832) has multi-item subscales corresponding to two dimensions: (1) Cognitive trust (three items; an example item is as follows: “I think online physicians will treat his/her work with professionalism and dedication”); (2) Affective trust (four items; an example item is as follows: “I think online physicians will treat me like a friend, and that we will be free to share our thoughts, feelings and hopes with each other”).

**Intention to use HIT (T2):** An eight-item scale (Cronbach-α = 0.803) was adapted from Li D. et al. (2020) and Slepchuk et al.'s (2021) measures. An example item was “If I am sick, I will choose an internet hospital for online inquiry.”

**Control variables (T1):** In our statistical analysis, we controlled for gender, age, education level, and pay because these four variables are widely used demographic variables in management research and may be related to the study variables, including communication ambiguity, communication effort, trust, and behavioral intention.

## Results

### Confirmatory Factor Analysis

We conducted confirmatory factor analysis *via* Amos 26 to assess the discriminant validity of the measurement model. The results in Table 1 indicated that the hypothesized five-factor model fits the data well (χ^2^/*df* = 1.939, RMSEA = 0.051, SRMR = 0.049, CFI = 0.931, IFI = 0.932). The results provided support for taking the five constructs as distinctive variables, and the five-factor model was thus retained for substantial hypothesis tests.

**Table 1:** Confirmatory factor analysis model fit results.

**Models**	**χ^2^**	***df***	**χ^2^/*df***	**RMSEA**	**SRMR**	**CFI**	**IFI**
*Five-factor model:*							
The hypothesized five-factor model	548.599	283	1.939	0.051	0.049	0.931	0.932
*Four-factor model:*							
Combining HITMN and CA	852.459	293	2.909	0.073	0.057	0.855	0.856
Combining HITMN and CE	770.091	293	2.628	0.067	0.055	0.876	0.877
Combining HITMN and trust	897.125	293	3.062	0.076	0.061	0.843	0.844
Combining CA and CE	776.814	293	2.651	0.068	0.056	0.874	0.875
*Three-factor model:*							
Combining HITMN, CA, and CE	885.262	296	2.991	0.074	0.058	0.847	0.848
Combining CA, CE, and trust	932.386	296	3.150	0.077	0.063	0.835	0.836
*Two-factor model:*							
Combining HITMN, CA, CE, and trust	1,057.447	298	3.548	0.084	0.066	0.802	0.804
Combining HITMN, CE, trust, and BI	1,107.444	298	3.716	0.087	0.070	0.789	0.791
*One-factor model:*							
Combining all variables	1,234.092	299	4.127	0.093	0.073	0.757	0.758

*HITMN, HIT media naturalness; CA, Communication ambiguity; CE, Communication effort; BI, Intention to use HIT*.

*χ^2^, Chi-square; df, degrees of freedom; RMSEA, root mean square error of approximation; SRMR, standardized root mean square residual; CFI, comparative fit index; IFI, incremental fit index*.

### Test of Common Method Bias

Due to that all data were collected from a single source, the potential impacts of common method bias should be examined. We followed the suggestions of Podsakoff et al. (2012) to test the potential common method bias. As shown in Table 1, the hypothesized five-factor model (χ^2^/*df* = 1.939, RMSEA = 0.0051, SRMR = 0.049, CFI = 0.931, IFI = 0.932) demonstrates better model fit indexes than the one-factor model (χ2/*df* = 4.127, RMSEA = 0.093, SRMR = 0.073, CFI = 0.757, IFI = 0.758). Moreover, no other alternative two-factor models, three-factor models, or four-factor models reveal better model fit. Thus, the common method bias issue was unlikely to have distorted the results of this study.

### Hypothesis Test

Table 2 shows the descriptive statistics, correlations, and reliabilities of all the variables in our study. All internal reliabilities were above 0.80, except for communication effort. As expected, HIT media naturalness was significantly correlated with communication ambiguity (*r* = −0.584, *p* < 0.01), communication effort (*r* = 0.629, *p* < 0.01), trust (*r* = 0.537, *p* < 0.01) and behavioral intention (*r* = 0.535, *p* < 0.01). Communication ambiguity was significantly correlated with trust (*r* = −0.602, *p* < 0.01) and behavioral intention (*r* = −0.401, *p* < 0.01). Communication effort was significantly correlated with trust (*r* = 0.605, *p* < 0.01) and behavioral intention (*r* = 0.543, *p* < 0.01). Trust was significantly correlated with behavioral intention (*r* = 0.610, *p* < 0.01). These results provide preliminary support for our hypotheses.

**Table 2:** Means, standard deviations, correlations, and reliabilities of studied variables.

	**Mean**	**SD**	**1**	**2**	**3**	**4**	**5**	**6**	**7**	**8**	**9**
1. Gender	1.609	0.489	_								
2. Age	3.422	0.204	−0.106^*^	_							
3. Education	3.931	0.595	0.031	−0.004	_						
4. Pay	2.080	0.905	−0.067	0.271^**^	0.258^**^	_					
5. HIT media naturalness	3.995	0.666	0.004	0.145^**^	0.071	0.330^**^	(0.828)				
6. Communication ambiguity	1.812	0.745	−0.106^*^	−0.092	−0.132^*^	−0.114^*^	−0.584^**^	(0.826)			
7. Communication effort	4.183	0.637	−0.017	0.136^**^	0.070	0.273^**^	0.629^**^	−0.650^**^	(0.757)		
8. Trust	4.271	0.471	0.037	0.154^**^	−0.042	0.160^**^	0.537^**^	−0.602^**^	0.605^**^	(0.832)	
9. Intention to use HIT	4.161	0.482	−0.034	0.110^*^	0.069	0.288^**^	0.535^**^	−0.401^**^	0.543^**^	0.610^**^	(0.803)

*N = 361*.

* *p < 0.05,*

** *p < 0.01. Cronbach's alphas are shown in parentheses along the diagonal. SD, standard deviation.*

*Gender: 1 = male, 2 = female; Education level: 1 = primary school, 2 = middle school, 3 = junior college, 4 = undergraduate, 5 = master's degree or above; Pay: 1 = less than 100,000 yuan/year, 2 = 100,000–200,000 yuan/year, 3 = 200,000–300,000 yuan/year, 4 = 300,000–400,000 yuan/year, 4 = 400,000–500,000 yuan/year, 6 = more than 500,000 yuan/year; Age is in years*.

**Figure 2:**
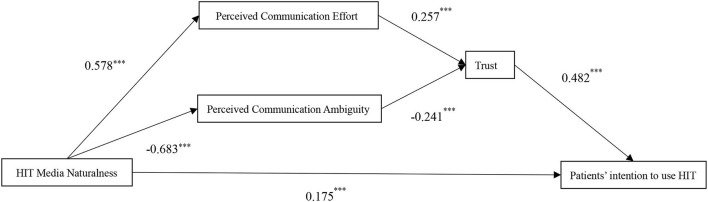
Results of serially mediation model estimation. *N* = 361. ***p < 0.001. The path coefficient is unstandardized regression estimation.

We conducted path analysis with composites of research variables, using Amos 26 to examine our hypothesis. Item scores were calculated as the average for each variable. The path model included all direct paths from independent variable (HIT media naturalness) to mediating variables (communication ambiguity, communication effort, and trust toward online physicians), all direct paths from mediating variables to dependent variable (intention to use HIT), as well as the direct path from independent variable to dependent variable. Furthermore, indirect effects were tested *via* bootstrapping. After controlling for gender, age, education level and pay, the results of all paths were shown in Table 3. We also present the significant direct unstandardized path estimates in Figure 2.

**Table 3:** Results of path analyses.

**Path**	**Coefficient**	**SE**	**CR**	**95% CI intervals**	
				**Lower level**	**Upper level**
HITMN → BI	0.175^***^	0.035	5.022	0.086	0.276
HITMN → CE	0.578^***^	0.041	13.97	0.458	0.693
HITMN → CA	−0.683^***^	0.05	−13.788	−0.841	−0.52
CE → Tr	0.257^***^	0.039	6.535	0.112	0.439
CA → Tr	−0.241^***^	0.033	−7.304	−0.357	−0.109
Tr → BI	0.482^***^	0.048	10.106	0.319	0.634
Gen → CE	−0.018	0.054	−0.329	−0.119	0.079
Age → CE	0.002	0.004	0.409	−0.006	0.01
Edu → CE	0.012	0.045	0.267	−0.097	0.142
Pay → CE	0.047	0.032	1.454	−0.013	0.111
Gen → CA	−0.147^*^	0.064	−2.291	−0.287	−0.026
Age → CA	−0.005	0.005	−1.079	−0.015	0.006
Edu → CA	−0.149^*^	0.054	−2.738	−0.319	−0.008
Pay → CA	0.101^*^	0.039	2.609	0.016	0.195
Gen → Tr	0.014	0.038	0.379	−0.054	0.093
Age → Tr	0.004	0.003	1.517	0	0.009
Edu → Tr	−0.1^**^	0.032	−3.101	−0.174	−0.03
Pay → Tr	0.022	0.022	0.982	−0.022	0.063
Gen → BI	−0.05	0.039	−1.29	−0.134	0.028
Age → BI	−0.004	0.003	−1.495	−0.013	0.004
Edu → BI	0.03	0.033	0.917	−0.042	0.103
Pay → BI	0.071^***^	0.023	3.036	0.03	0.116

*HITMN, HIT media naturalness; CE, communication effort; CA, communication ambiguity; Tr, Patients' trust toward online doctors; BI, intention to use HIT*.

* *p < 0.05,*

** *p < 0.01,*

*** *p < 0.001*.

As shown in Table 3 and Figure 2, HIT media naturalness was positively related to patients' intention to use HIT (β = 0.175, *p* < 0.001). Thus, Hypothesis 1 was supported.

Hypothesis H2 proposed that HIT media naturalness will be positively related to patients' perceived online communication effort (Hypothesis H2a), and negatively related to communication ambiguity (Hypothesis H2b). As shown in Figure 2, there revealed a positive association between HIT media naturalness and patients' perceived online physician's communication effort (β = 0.578, *p* < 0.001), and a negative association between HIT media naturalness and communication ambiguity (β = −0.683, *p* < 0.001). Thus, Hypotheses H2a and H2b received the support.

The empirical results also showed that patients' perceived online physician's communication effort was positively related to patients' trust toward online physicians (β = 0.257, *p* < 0.001). And patients' perceived communication ambiguity was negatively related to patients' trust toward online physicians (β = −0.241, *p* < 0.001). Thus, Hypotheses H3a and H3b were supported.

Moreover, patients' trust toward online physicians was positively related to patients' intention to use HIT (β = 0.482, *p* < 0.001), which means that the higher the patients' trust toward online physicians, the higher is the patients' intention to use HIT. Thus, Hypothesis 4 was supported.

To test Hypothesis H5a and H5b, we tested the significance of the indirect effects *via* bootstrapping analysis by using Amos 26. The results of the indirect effects analyses were presented in Table 4.

**Table 4:** Mediation effect estimated by bootstrap methods and 95% CI intervals.

**Path**	**Estimates**	**95% CI intervals**	
		**Lower level**	**Upper level**
Total indirect effect	0.151	0.09	0.223
**Separate indirect effect**			
Path 1: HITMN->CE->Tr->BI	0.072	0.031	0.137
Path 2: HITMN->CA->Tr->BI	0.079	0.033	0.141

*HITMN, HIT media naturalness; CE, communication effort; CA, communication ambiguity; Tr, Patients' trust toward online doctors; BI, intention to use HIT*.

As expected, patients' perceived online physicians' communication effort and trust toward online physicians significantly acted as serial mediators between HIT media naturalness and patients' intention to use HIT [indirect effect = 0.072, *p* < 0.001, 95% CI intervals = (0.031, 0.137)], as its confidence interval did not include zero. Additionally, patients' perceived communication ambiguity and trust toward online physicians serially mediated the relationship between HIT media naturalness and patients' intention to use HIT significantly [indirect effect = 0.079, *p* < 0.001, 95% CI intervals = (0.033, 0.141)], as its confidence interval did not include zero. Thus, Hypothesis H5a and H5b received support.

## Discussion

### Conclusions

Drawing on media naturalness theory, this study aims to test the potential mechanisms on the influence of HIT media naturalness on patients' intention to use HIT. Based on a time-lagged survey of 361 participants, the empirical findings revealed that HIT media naturalness was positively related to patients' intention to use HIT; the relationship between HIT media naturalness and patients' intention to use HIT was serially mediated through two paths, that is, (1) patients' perceived online physicians' communication effort and trust toward online physicians and (2) patients' perceived communication ambiguity and trust toward online physicians.

### Theoretical Contribution

This study makes several theoretical contributions. First, by drawing on media naturalness theory, this study enriches the research on the intention to use HIT. The previous studies have demonstrated that the effectiveness of online healthcare platforms (Yang et al., 2018), patients' perceived fairness (Le et al., 2019), patients' prosocial motivation (Kokkoris and Kamleitner, 2020), patients' attitudes toward behavior (Li D. et al., 2020) and trust and interaction between the doctors and patients (Yang et al., 2021) play important roles in patients' intention to use HIT. Our study found that the HIT media naturalness promotes patients' intention to use HIT through two chain mediations. To our knowledge, this is the first attempt to explore how HIT media naturalness influences patients' intention to use HIT. This finding thus enriches the literature on patients' intention to use HIT by introducing media naturalness theory.

Second, this study expands the media naturalness theory to the field of HIT behavioral intention. Despite the rich development of media naturalness theory, there has not been adequate related research in the field of trust and intention to use HIT. Yang et al. (2021) suggested that future research could investigate cognitive mechanisms, such as exploring the influence of trust on online health based on media naturalness theory. Based on the extension of the media naturalness theory, Zahedi et al. (2016) first established an augmented virtual doctor office (AVDO), which simulates the naturalness of face-to-face visits through an immersive environment. In response to the call from those studies, we further extent the media naturalness theory to online healthcare research through empirical studies.

Third, this study contributes to the existing literature by enriching the antecedent of trust from the perspective of physician-patient communication. The previous studies have mainly focused on the patients' trust toward online physicians based on interpersonal relationships or doctors' characteristics. For example, online physicians' characteristics (such as benevolence, competence, integrity, etc.) would affect patients' trust in doctors (Li et al., 2018; Wan et al., 2020; Cantarutti and Pothos, 2021; Gong et al., 2021). Based on the media naturalness theory, our study investigates how media naturalness would affect the elements of physician–patient communication, then affecting patients' trust toward online physicians. Our empirical findings show that patients' perceived online physicians' communication effort and patients' perceived communication ambiguity affect their trust toward online physicians, which enriches the research of trust from the perspective of medium characteristics and communication process.

### Practical Contribution

Our study also provides several practical contributions for HIT service. On the one hand, a more natural medium would promote patients' intention to use HIT. When accessing a platform, patients expect to find a useful channel, whose functions contribute to health information search, doctor selection, health consultation, and rating (Yoo et al., 2020). The empirical results of this study prove that media naturalness promotes patients' intention to use HIT. Therefore, a more natural medium can attract more users. In the process of establishing an online healthcare platform, designers could try to create more naturalness, which makes users feel like being with others (Zahedi et al., 2016).

On the other hand, adequate communication between doctors and patients is conducive to promoting patients' trust in online physicians, and improving patients' intention to use HIT. Patients often complain that physicians do not listen to their concerns, care about their problems, or provide enough information about their treatment (Hickson et al., 1994). Our study found that patients' perceived online physicians' communication effort promote their trust toward online physicians, while patients' perceived communication ambiguity reduces their trust toward online physicians. This suggests that in practical application, online physicians and platforms should actively take various measures to improve patients' trust, especially patient-centered communication skills.

### Limitation and Future Direction

Although this study has provided both theoretical and practical contributions, there are also some limitations, which are worthy of further research and improvement. Specific research limitations and future research directions are discussed in the following paragraphs.

First, although this study discusses the influence of media naturalness on the intention to use HIT, we only apply the survey method to obtain data and test research hypotheses. However, the questionnaire method can only verify the correlation between research variables. To better test the possible causality in the hypothesis model, future research could use experimental methods for verification. For example, an experimental group and a control group could be established to study whether the experimental group would have higher intentions to use HIT when media naturalness is enhanced.

Second, the studied sample of this research may limit our understanding of users' intention to use HIT. Specifically, the majority of our participants (91.7%) are aged between 20 and 40 years. This is because although the patients are more likely to be lower aged or elder, the younger patients in targeted patient communication groups might be more willing to participate in the online survey (Li D. et al., 2020). Thus, failing to incorporate elderly users has always been a research gap in existing HIT-related studies (e.g., Hong et al., 2019; Li D. et al., 2020; Wan et al., 2020). The understanding and demand of HIT may vary considerably from elder users to young users. For example, studies have indicated that although elder people may show greater needs for healthcare services, they are less likely to use HIT (Slepchuk et al., 2021). This may be due to their physical disabilities, such as poor vision, cognitive disabilities, and motor skill limitations (Niehaves and Plattfaut, 2014), as well as their unwillingness to adopt HIT stemming from mistrust, high risk perceptions, and strong desire for privacy (Fox and Connolly, 2018). Therefore, failing to incorporate those elderly uses would make it difficult to generalize our research findings. We thus encourage future research to consider a wide range of people of different ages and experiences to verify and expand our research model.

Third, the HITs which respondents used in this study may prevent us from validating the research findings. Although we measured the respondents' perceived HIT media naturalness based on their past online healthcare experiences, which were not focusing on several kinds of HITs, our respondents commonly use some popular online health platforms in China (e.g., haodf.com, dingxiangyisheng.com, chunyuyisheng.com etc.). This might limit the validity of our research findings within specific HIT contexts. Thus, our findings should be viewed with caution when generalizing them to a broader context. In addition, as we focus on online health consultation in China, our conclusions may not be fully applicable to other cultures, because individuals raised in different cultural environments usually have different information processing schemas (Kock, 2002, 2005). It is necessary to test the validity of our conclusions in other cultures and modify our suggestions accordingly.

Fourth, given that self-reports were collected for all variables, this study did approach common method variance (CMV) influences with confirmatory factor analysis (CFA) procedure. Although it passed the homogeneity test eventually, potential CMV may still interfere with causality. Hence, the researchers can further enrich our results by obtaining measures from different sources in the future (Podsakoff et al., 2012). For example, the independent variable HIT media naturalness could be measured through survey, and the dependent variable patients' intention to use HIT could be obtained through objective data.

Finally, based on the media naturalness theory, our study investigated the mediating mechanism of media naturalness on the intention to use HIT. Future research could explore the boundary conditions of media naturalness. For example, patients with different personalities or ages may perceive different degrees of communication ambiguity even when faced the same level of media naturalness.

## Data Availability Statement

The original contributions presented in the study are included in the article/supplementary material, further inquiries can be directed to the corresponding author.

## Author Contributions

All authors contributed to the article and approved the submitted version.

## Funding

This research was supported by the National Natural Science Foundation of China (Nos. 71902164 and 71902148), China Postdoctoral Science Foundation (No. 2019M660243), Sichuan University (No. 2020CXQ23), and the Fundamental Research Funds for the Central Universities (Nos. skbsh2020-25 and 2022ZYSX009).

## Conflict of Interest

The authors declare that the research was conducted in the absence of any commercial or financial relationships that could be construed as a potential conflict of interest.

## Publisher's Note

All claims expressed in this article are solely those of the authors and do not necessarily represent those of their affiliated organizations, or those of the publisher, the editors and the reviewers. Any product that may be evaluated in this article, or claim that may be made by its manufacturer, is not guaranteed or endorsed by the publisher.

## Appendix: Measures of Hit Media Naturalness

Please recall your past experience of online health service, would you agree that (1) the communication between you and your online physicians made you feel that you were co-located in the same physical space. (2) The communication between you and your online physicians had high levels of synchronicity, which enabled immediate and spontaneous responses to stimuli. (3) The interaction between you and your online physicians enabled you to identify and convey facial expressions. (4) The interaction between you and your online physicians enabled you to identify and convey body language. (5) The communication between you and your online physicians enabled you to convey natural speech.
